# Interactions among Endothelial Nitric Oxide Synthase, Cardiovascular System, and Nociception during Physiological and Pathophysiological States

**DOI:** 10.3390/molecules27092835

**Published:** 2022-04-29

**Authors:** Niribili Sarmah, Andromeda M. Nauli, Ahmmed Ally, Surya M. Nauli

**Affiliations:** 1 Arkansas College of Osteopathic Medicine, Fort Smith, AR 72916, USA; nsarmah@acheedu.org; 2 Department of Biomedical Sciences, Western Michigan University Homer Stryker M.D. School of Medicine, Kalamazoo, MI 49008, USA; andromeda.nauli@med.wmich.edu; 3 Department of Biomedical and Pharmaceutical Sciences, Chapman University, Irvine, CA 92618, USA; 4 Department of Medicine, University of California, Irvine, CA 92697, USA

**Keywords:** nociception, periaqueductal gray matter, hypertension, atherosclerosis, autonomic nervous system, endothelial cells, glutamate, GABA, blood pressure, heart rate, reactive oxygen species, antioxidants, hypothalamus

## Abstract

Nitric oxide synthase (NOS) plays important roles within the cardiovascular system in physiological states as well as in pathophysiologic and specific cardiovascular (CV) disease states, such as hypertension (HTN), arteriosclerosis, and cerebrovascular accidents. This review discusses the roles of the endothelial NOS (eNOS) and its effect on cardiovascular responses that are induced by nociceptive stimuli. The roles of eNOS enzyme in modulating CV functions while experiencing pain will be discussed. Nociception, otherwise known as the subjective experience of pain through sensory receptors, termed “nociceptors”, can be stimulated by various external or internal stimuli. In turn, events of various cascade pathways implicating eNOS contribute to a plethora of pathophysiological responses to the noxious pain stimuli. Nociception pathways involve various regions of the brain and spinal cord, including the dorsolateral periaqueductal gray matter (PAG), rostral ventrolateral medulla (RVLM), caudal ventrolateral medulla, and intermediolateral column of the spinal cord. These pathways can interrelate in nociceptive responses to pain stimuli. The alterations in CV responses that affect GABAergic and glutamatergic pathways will be discussed in relation to mechanical and thermal (heat and cold) stimuli. Overall, this paper will discuss the aggregate recent and past data regarding pain pathways and the CV system.

## 1. Introduction

Endothelial nitric oxide synthase (eNOS) is one of the isoforms of nitric oxide synthase (NOS). Endothelial NOS is highly expressed in vascular endothelial cells. The biochemical reaction to produce nitric oxide is shown below:

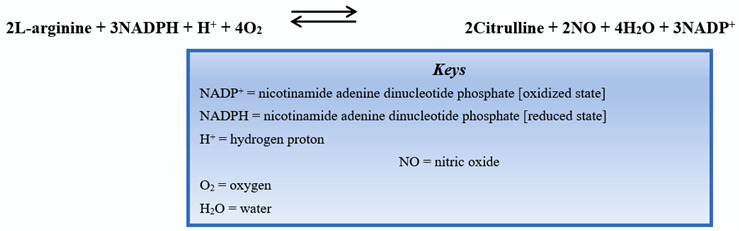



**Figure 1:**
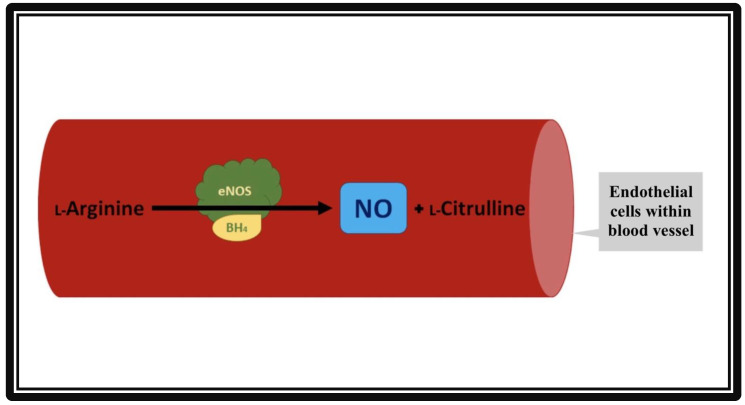
Formation of nitric oxide (NO) from L-Arginine in the presence of co-factor tetrahydropbiopterin (BH_4_) and endothelial nitric oxide synthase (eNOS). The vascular endothelial cells produce NO by utilizing eNOS. The NO then diffuses from vascular endothelia to vascular smooth muscle cells. Inside the smooth muscles, NO activates guanylate cyclase, which converts GTP to cGMP. cGMP relaxes the vascular smooth muscle cells, resulting in vasodilation.

Discovered in 1991 [1], the eNOS enzyme was found to be responsible for the production of nitric oxide (NO), an essential compound in vasodilation responses and a critical regulator of cardiovascular (CV) homeostasis [2]. In addition to its action in vascular endothelial cells, eNOS is also active in hippocampal neurons as well as postganglionic sympathetic and dorsal root ganglion [3]. Nitric oxide increases the activity of guanylyl cyclase, an enzyme responsible for producing cyclic guanosine monophosphate (cGMP) that promotes smooth muscle relaxation. Endothelial NOS maintains a healthy CV system (CVS), partly by regulating and maintaining vascular tone: migration, production, and maturation of cells; leukocyte adhesion; and platelet aggregation [4]. A schematic of NO production by the endothelial cells of the vasculature is shown in Figure 1.

### 1.1. Nociception

This manuscript’s primary focus is to elaborate the roles of eNOS in relationship to nociception and CV system. Nociception is described as a subjective sensory and emotional experience that is accompanied by some degree of tissue damage. Stimuli of a painful nature may result from different inputs including mechanical, chemical, or thermal origins. Mechanical responses include injury such as a laceration to the skin [5], while chemical stimuli may include events such as exposure to a “hot” gustatory stimulus, such as capsaicin, on the tongue [6]. In addition, thermal stimuli include contact with extreme heat or extreme cold [7]. These phenomena may stimulate sensory pain receptors that will relay signals to different areas of the brain via afferent fibers in the peripheral and central nervous system. These signals are integrated by sensory integration centers involving the medulla oblongata, periaqueductal gray matter (PAG), hypothalamus, and thalamus [8]. The aforementioned relay pathways are relevant to the physiological role played by eNOS. Stimulation of nociceptors may activate the eNOS pathway via autonomic nervous system-mediated inflammation and vasodilation cascades [9]. The CVS plays a significant role in the appropriate execution of such cascades. Accordingly, we will discuss these pathways in depth, including the function of eNOS in relation to CV pathologies, such as hypertension and atherosclerosis.

### 1.2. Cardiovascular System

The CV system is a complex arrangement of arteries, capillaries, veins, and a central organ or the heart that works systematically to circulate blood throughout the body. It is a critical system that drives the transportation of vital resources and signals to different areas in the body, including oxygen, carbon dioxide, vitamins, minerals, neurotransmitters (for platelet aggregation), insulin, and NO [10]. The eNOS isoenzyme can interact with the cardiovascular system by regulating signal relay and resource allocation. However, the exact mechanism delineating this relationship with nociception remains unknown. For example, reduced function or bioavailability of the eNOS isoenzyme is linked to increased risk of essential hypertension, preeclampsia, diabetic nephropathy, retinopathy, migraine, and erectile dysfunction in humans [11]. As noted, reduced eNOS expression produces several deleterious effects; however, eNOS upregulation can result in homeostatic dysregulation as well [12]. Overexpression in the rostral ventrolateral medulla (RVLM) is positively correlated with hypotension and bradycardia. Both pathologies are preceded by altered neurotransmitter levels, namely increased levels of inhibitory gamma amino-butyric acid (GABA) and decreased levels of excitatory glutamate [12]. Upregulation of eNOS may be a result of stress caused by the release of acetylcholine (ACh), bradykinin, histamine, and 17β-estradiol-mediated phosphorylation [13]. The following sections will describe and present the scientific literature that is pertinent to the complex relationships among nociception, CVS, and NO.

## 2. Endothelial Nitric Oxide Synthase and Nociception

Nociception, or more commonly known as pain, is a sensation that also involves an emotional component. The International Association for the Study of Pain (IASP9) offers the following definition of pain: “An unpleasant sensory and emotional experience associated with actual or potential tissue damage or described in terms of such damage” [14]. We have previously published an extensive review regarding the role of nNOS isoform on CV responses elicited during mechanically, heat-, and cold-induced changes in mean arterial pressure (MAP) and heart rate (HR) using a model of anesthetized rat [9]. Briefly, the alterations in CV responses due to pain are dependent on the type of pain, intensity of pain, involvement of specific nociceptors, neural pathways, and the involvement of various neurotransmitters or receptors [15]. It has been widely studied that the eNOS system interacts with the ventrolateral medullar (VLM) and PAG to modulate pain. Moreover, several brain regions such as the RVLM, the caudal ventrolateral medulla (CVLM), and the dorsolateral periaqueductal gray matter (dlPAG) play differential roles in integrating and modulating CV responses during pain [13]. Manipulating NO concentrations within the dlPAG or medulla oblongata affects the glutamatergic and GABAergic pathways during nociceptive experiences triggered by thermal (heat/cold) stimuli but not mechanical stimuli or pinch/pressure [16]. Past studies demonstrate that dysregulation of all NOS isoforms (nNOS, eNOS, inducible NOS (iNOS)) can significantly alter the experience of pain. For example, overproduction of NO by all NOS isoforms may result in neuroprotective or neurotoxic impacts mediated through the generation of reactive oxygen waste products (peroxynitrite) [17]. Subsequent chain reactions with carbon dioxide (CO_2_) can induce the destruction of neurons by way of oxidizing lipids, denaturing polypeptides, and destabilizing structural bases in DNA [17].

**Figure 2:**
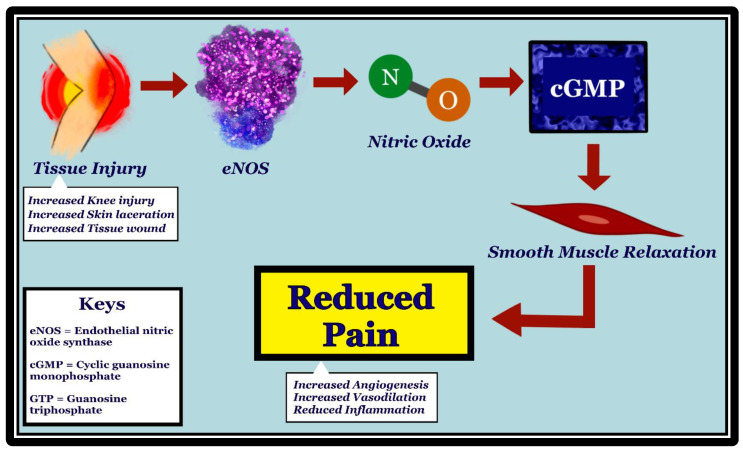
Mechanism of how initial tissue injury leads to pain reduction through activation of eNOS, production of nitric oxide (NO), and increase in cGMP. eNOS is stimulated in vascular endothelia by tissue injury, such as knee injury, skin laceration, and wounds. NO, which is produced by eNOS, diffuses to the vascular smooth muscle cells. Upon stimulation by NO, guanylate cyclase converts GTP to cGMP, resulting in vascular smooth muscle relaxation. Vasodilation has been shown to be associated with pain reduction, possibly through reduced inflammation and increased angiogenesis.

While substantial evidence regarding the involvement of nNOS and iNOS in producing neuropathic pain has been uncovered, the relationship between eNOS and nociception remains unexplored due to their complicated and irregular interactions [9]. Undoubtedly, NO is implicated in both pro-nociception as well as anti-nociception [18]. Several studies find inconsistent results regarding the effect of arginine bioavailability on the pain status of the participants. One such study evaluates this relationship in both chronic and acute pain conditions related to sickle cell disease (SCD) [19]. The reduced NO from SCD-related hemolysis and oxidative stress is noted to result in endothelial dysfunction during experiences of vaso-occlusive pain [19]. This dynamic is consistent throughout varying participant demographics, including adults and children. Another study appraises the eNOS-nociceptive pathway in rats with chronic post-ischemia pain (CPIP) through norepinephrine-induced nociception and vasoconstrictor hypersensitivity [20]. Chronic post-ischemia pain (CPIP+) rats and control rats received intradermal injection of either vasopressin or the eNOS inhibitor (N5)-(1-Iminoethyl)-*L*-ornithine dihydrochloride (*L*-NIO), and their nociceptive responses were compared [20]. Nociceptive responses were produced at significant levels (*p* < 0.01) in rats injected with *L*-NIO, suggesting that eNOS-facilitated vascular dilatation may attenuate pain responses [20]. Please see Figure 2 for the proposed mechanism.

Using a chronic constriction injury (CCI) rat model, Chu et al. showed that atorvastatin, a cholesterol-lowering drug, could reduce nociceptive sensitization and thermal hyperalgesia of peripheral neuropathy. It improved the inflammatory activity through reductions in cyclooxygenase-2 (COX-2) and iNOS. In addition, atorvastatin also increased neuroprotective factors that elicit positive responses in angiogenesis, including eNOS, vascular endothelial growth factor (VEGF), and (phosphorylated) protein kinase B (pAkt/Akt). However, this angiogenic property was not observed in eNOS knock-out mice, suggesting the importance of the eNOS-associated signal cascade in promoting vascular growth during nociceptive events [21]. This relationship is in alignment with what we know thus far of the VEGF signaling pathway, which has been shown to upregulate expression of Akt and eNOS [22]. Thus, improving vascular permeability can, in turn, aid in the recovery process of injured nervous tissue [23]. The protective role played by eNOS is further supported by its neuroprotective capabilities under conditions of ischemia or shear stress, positing the role of eNOS in earlier states of inflammatory nociception versus a neurotrophic role in later states [3,24]. Specifically, eNOS serves as a messenger to peripheral and central afferent neuronal sensitization following noxious stimuli [25]. Moreover, cluster of differentiation 31 (CD31) immunopositivity assay is shown to increase staining in complete Freund’s adjuvant (CFA) subject vessels, a comparable effect to eNOS that might indicate an interrelation since CD31 can modulate eNOS activity during shear stress via association or dissociation [3]. Evidence in favor of this finding is endorsed by an analysis of the role of chronic prostatitis/pelvic pain syndrome (CP/CPPS) and its relationship with endothelial dysfunction, specifically the expression of eNOS and cGMP levels in experimental autoimmune prostatitis (EAP), a rat model of CP/CPPS [26]. CP/CPPS is an inflammatory condition involving persistent pain and discomfort in the pelvic and genital areas.

## 3. Endothelial Nitric Oxide Synthase and the Cardiovascular System

In the CV system, eNOS mediates vasodilation via the production of NO. In response to a surplus of L-arginine, the severity of acute episodic pain related to sickle cell anemia (SCD), a chronic anemia exacerbated by CV complications, was reduced [19]. Of note, SCD patients have arginine deficiency. The cell-free hemoglobin from the lysed sickled erythrocytes consume nearby NO via the reaction shown below [27]:

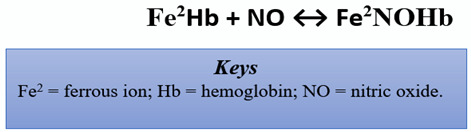



This reaction is an eradicant pathway for NO bioactivity. It proceeds at a rapid rate of 6–8 × 10^7^ per molar per second, rendering NO into the dead-end product, nitrate [28]. Thus, reduced NO is conceivably tied to vaso-occlusive pain and nociceptive processing. Herein, the reduced bioavailability of arginine in such patients is linked with experiences of acute pain, vaso-occlusive pain, endothelial dysfunction, pulmonary complications, risk of leg ulcers, and early mortality [29]. However, the specific mechanism remains unclear, as other studies corroborate increased pain in association with elevated levels of arginine [30].

Individuals carrying the thymine-cytosine and cytosine-cytosine (TC/CC) genotypes and the C allele for the g.-786T > C polymorphisms (eNOS) were more likely to respond better to enalapril, an angiotensin-converting enzyme (ACE) inhibitor. The polymorphism may be responsible for a more robust NO production, conferring the individuals a better response to the antihypertensive drug [31]. Peroxisome proliferator-activated receptor gamma coactivator-1alpha (PGC-1α) has also been shown to enhance NO production and reduce blood pressure. However, it increased NO production not by altering eNOS expression, but by inhibiting the uncoupling of eNOS dimers [32].

Several studies aim to explore the roles of transient receptor potential vanilloid type 1 (TRPV1) on NOS activation and its subsequent downstream effects of CV-specific angiogenesis in primary nociceptive sensory neurons. TRPV1 is mainly distributed throughout vascular endothelial cells, smooth muscle cells, and perivascular nerve cells in the cardiovascular system. It is responsive to inflammatory and noxious stimuli and can serve as a blood pressure mechanoreceptor within vasculature [33]. In addition, NO-dependent processes as a result of TRPV1 stimulation are a vital facet in the creation of new blood vessels and wound-healing, two factors that are important in countering the effects of cardiovascular disease such as arteriosclerosis or ischemic heart disease [34]. Activation of TRPV1 in endothelial cells promotes Ca^2+^-dependent signaling of phosphatidylinositol-3-kinase (PI3K), Akt, and Ca^2+^/calmodulin-dependent protein kinase II (CaMKII), which subsequently leads to increases in TRPV1-eNOS complex formation, eNOS activation, and NO production. eNOS and TRPV1 knockouts displayed reduced angiogenesis, increased atherosclerotic lesions, and reduced phosphorylation of eNOS and Akt [35].

Previously, it has been shown that the reduced atherosclerosis through uncoupling activity results in reduction of NO [13]. Studies explore oxidized low-density lipoprotein (Ox-LDL) as a cytotoxic agent that can impair endothelial cells and exacerbate the progression of atherosclerosis. This mechanism is an advancement of oxLDL-mediated inflammation that consequently decreases peroxisome proliferator-activated receptor gamma (PPARγ) endothelium protection, a factor that typically participates in eNOS expression through AMP-activated protein kinase (AMPK) and lectin-like oxidized low-density lipoprotein receptor-1 (LOX-1) signal cascades [36]. Inactivation of this pathway can cause detrimental fluctuation of atherosclerotic plaques and vascular malfunction.

Sildenafil (Viagra^®^, Revatio^®^) is a phosphodiesterase type 5 inhibitor (PDE5 inhibitor) that induces vasodilation by preventing PDE5 from degrading cGMP. Sildenafil exerts its neuroprotective effect by inhibiting inflammation and demyelination in the cerebellum [37]. Sildenafil was capable of decreasing the expression of the pro-inflammatory cytokines interleukin-1-beta (IL-1β) and tumor necrosis factor alpha (TNF-α); decreasing glial fibrillation acidic protein (GFAP), nuclear factor kappa light chain enhancer of active B-cells (NFkB), inactive AMPK, iNOS; and increasing the anti-inflammatory cytokine IL-10 and nuclear factor alpha of kappa light polypeptide gene enhancer in B-cell inhibitor (IKβα) [37]. Based on this discovery, sildenafil likely induces its anti-inflammatory and neuroprotective effects through the modulation of AMPK–IKβα–NFκB signaling [37].

## 4. Interaction between Endothelial Nitric Oxide Synthase, Nociception, and the Cardiovascular System

Major inflammatory responses are due to an elevation of microvascular permeability for numerous chemicals, particularly macromolecules, which result in the manifestations of edema. An increase in edema may cause disturbed homeostasis and tissue dysfunction, especially in the endothelium of venules. As mentioned previously, NO is physiologically known to be an important signaling regulator of cardiovascular function, particularly the significance of its role in the control of microvascular permeability [38,39]. Experiments using isolated venules, single capillaries, and endothelial cells of eNOS knockout mice have demonstrated that increased eNOS activities will elevate microvascular permeability to macromolecules that may happen due to inflammatory responses or medications [40,41]. Definitive evidence to settle this initial controversy was obtained. Studies using engineered animals determine eNOS-derived NO causes hyperpermeability due to pro-inflammatory agents such as platelet-activating factor (PAF) and vascular endothelial growth factor (VEGF) both in vivo and in vitro [42,43]. The signaling cascades for several inflammatory agents or conditions also require the activation of protein kinase C (PKC) and eNOS, which are followed by synthesis of cGMP [44,45]. Finally, studies using isolated micro-blood vessels would show that stimulation of eNOS and subsequent production of cGMP leads to changes in permeability to both major and minor molecules (small and large solutes) [46]. Further physiological, pathophysiological, and molecular biology investigations will contribute to higher understanding of the knowledge that controls microvascular mechanism and will lead to the development of pharmacologic agents and treatment for systemic inflammation. The relationship among eNOS, CVS, and the CNS are previously mentioned in our recent review [13].

There have been more than 45 million studies that have focused on CVD and reduced eNOS activity. In short, various CVD risk factors such as hypertension, diabetes, insulin resistance, obesity and hyperlipidemia are because of reduced eNOS dysfunction [47]. It has been recognized that the eNOS mechanism of endothelial function is the prevention against atherogenesis cascades, such as vascular smooth muscle cell proliferation, inflammation, or thrombosis. As reported, eNOS dysfunction at a reduced production does not protect the normal physiologic and protective mechanisms of the endothelial cells [48,49], for example, if the endothelium is damaged with reduced eNOS, particularly for damaged endothelium and eNOS in coronary arteries subjected to angioplasty or percutaneous intervention, and metabolic toxins, such as free fatty acids (FFAs) and inflammatory cytokines, including IL-6 and TNF-α. Patients with hyperglycemia can also have advanced glycation products and other metabolic abnormalities that may affect eNOS-mediated intracellular signaling pathways; even the PI3K–Akt pathway and Akt downstream to PI3K that is inhibited by ipatasertib. In addition, any ROS can destroy vascular eNOS and prevent it from inducing vascular smooth muscle relaxation [50,51].

Regarding the drugs that are developed to modulate pain pathways, there have been more than 73 thousand research publications. In short, normal regulatory mechanisms of eNOS activity, such as calcium/calmodulin, caveolin, HSP90, palmitoylation, and myristoylation, can activate phosphorylation by AKT or AMP-activated protein kinase (AMPK) at Serine 1177. Other regulations of eNOS activity are based on the formation of redox-active species that cause dysfunctional phosphorylation by redox-active kinases at Thr495/Tyr657 [52,53]. REDOX alterations in eNOS disrupt eNOS activity and uncouple eNOS [52]. In addition, cGMP-degrading phosphodiesterases are activated by ROS [54,55]. Any changes of prostanoid synthesis/production to thromboxane can result in vasoconstriction and aggregatory phenotype [52].

Previously, we have reported that mechanically induced and heat-mediated thermal pain increase MAP and HR, while a cold-evoked thermal pain stimulus causes decreases in MAP and HR [56,57,58,59]. Although the link between the cardiovascular and nociceptive systems has been extensively studied, the precise mechanisms within the human brain that regulate this relationship are still unknown. Particularly, the plethora of diverse mechanisms, the interaction of numerous neurotransmitters/neuromodulators, specific regions of the brain, interaction among receptors, and different management or treatment protocols make the field of nociception research vast and more complex. Nevertheless, the CVS and nociception are interrelated and are displayed quite significantly by bodily homeostasis and defensive/withdrawal reactions including changes in cardiovascular hemodynamics. We have written a chapter in a book that describes numerous pain therapies that are prescribed for treatment of different variations of peripheral neuropathic pain in humans, including their side effects or toxicology on cardiovascular functions and the CVS [57].

**Figure 3:**
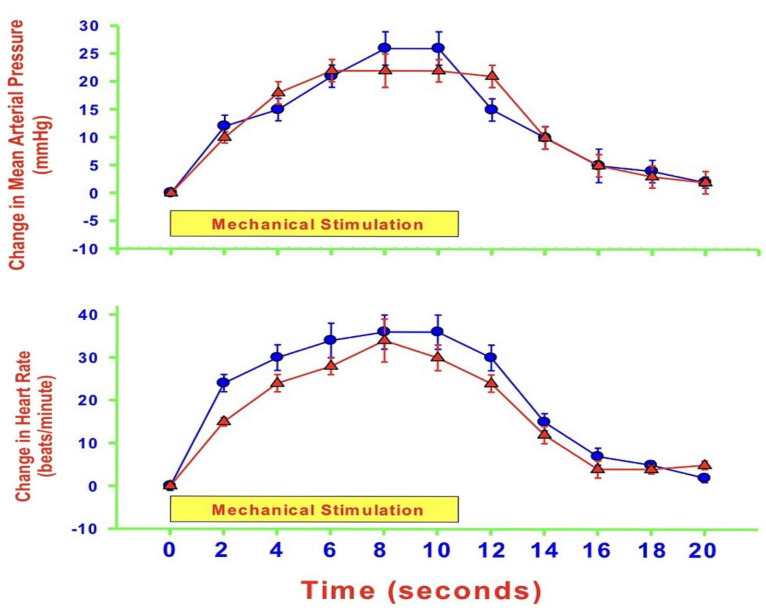
Average time-course changes in mean arterial pressure (MAP: upper panel) and heart rate (HR: lower panel) during 10 s hind paw mechanical stimuli 120 min after bilateral insertion of microdialysis probes into the dorsolateral periaqueductal gray matter (control: blue circles) and 30 min after microdialysis of L-N(5)-(1-iminoethyl) ornithine (L-NIO: 10 µM: red triangles; an endothelial nitric oxide synthase antagonist) into the same area of anesthetized rats. The Y-axes are changes in MAP or HR, and the yellow boxes represent the 10 s time of stimulation. Values represent means ± SEM (*n* = 8).

**Figure 4:**
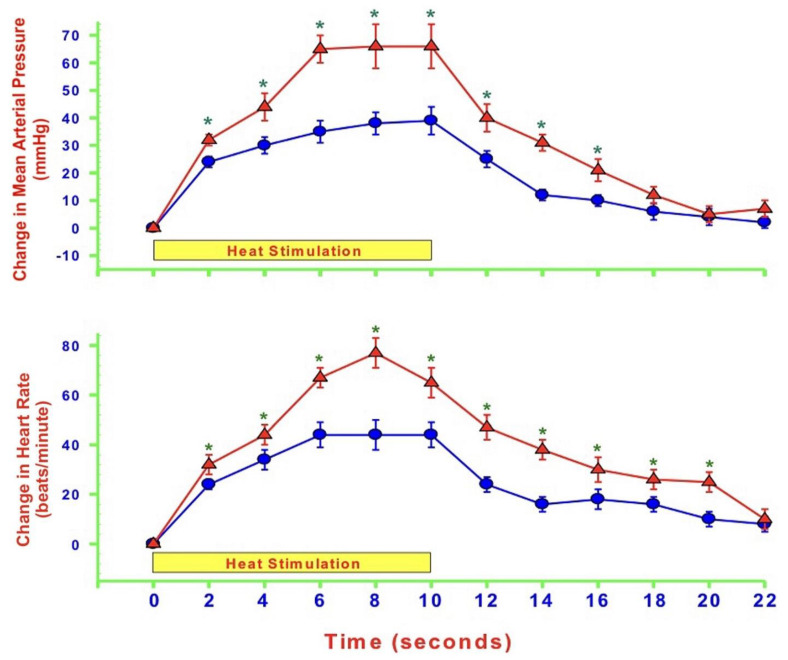
Average time-course changes in mean arterial pressure (MAP: upper panel) and heart rate (HR: lower panel) during 10 s hind paw heat-induced thermal stimuli 120 min after bilateral insertion of microdialysis probes into the dorsolateral periaqueductal gray matter (control: blue circles) and 30 min after microdialysis of L-N(5)-(1-iminoethyl)ornithine (L-NIO: 10 µM: red triangles; an endothelial nitric oxide synthase antagonist) into the same area of anesthetized rats. The Y-axes are changes in MAP or HR, and the yellow boxes represent the 10 s time of stimulation. Values represent means ± SEM (*n* = 8) and * *p* < 0.05 represents value versus control.

**Figure 5:**
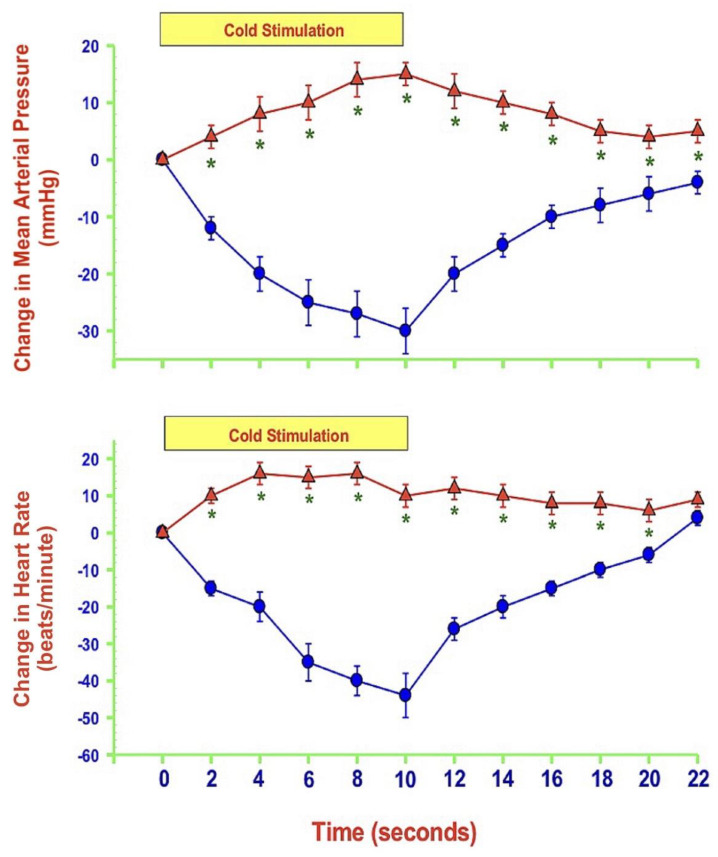
Average time-course changes in mean arterial pressure (MAP: upper panel) and heart rate (HR: lower panel) during 10 s hind paw immersion in a 10 °C cold-water bath 120 min after bilateral insertion of microdialysis probes into the periaqueductal gray matter (control: blue circles) and 30 min after microdialysis of L-N(5)-(1-iminoethyl)ornithine (L-NIO: 10 µM: red triangles; an endothelial nitric oxide synthase antagonist) into the dorsolateral periaqueductal gray matter of anesthetized rats. The Y-axes are changes in MAP or HR, and the yellow boxes represent the 10 s time of stimulation. Values represent means ± SEM (*n* = 8) and * *p* < 0.01 represents values versus control.

As mentioned, NO levels within the medulla oblongata or the dlPAG differentially modulate cardiovascular responses by altering glutamatergic and GABAergic neurotransmission during heat-induced, but not in response to mechanically induced nociceptive stimulation. One such example is that nNOS antagonism within the dlPAG potentiates cardiovascular responses during heat-induced thermal stimulation via increasing glutamate release and reducing GABA levels. nNOS, however, does not alter those cardiovascular responses and neurotransmission during mechanical nociception. Many reviews and studies using humans and animals demonstrate that NO, eNOS, iNOS, and nNOS play significant roles in neuronal excitability through a plethora of NO-related mechanisms involving nociception, particularly the eNOS protein and neuropathic pain [60]. A 2007 study compared the reactions to pain following intraplantar injection of CFA in wild-type (WT) and eNOS-deficient mice. The study found significant changes in the behavior reaction to pain in eNOS-deficient mice, including a rapid recovery from thermal hyperalgesia [61]. The first clinical trial that attempted to block all three NOS by using a non-selective NOS inhibitor, L-NG-methylarginine hydrochloride (546C88), shows that 546C88 is an effective drug for headache relief and can also improve the symptoms of sensory hypersensitivity [62]. Another human trial shows that the non-selective NOS inhibitor, NG-monomethyl-L-arginine hydrochloride (L-NMMA), provides significant relief from pain in patients with chronic tension-type headache [63]. Although eNOS has a role in the pathogenesis of migraine, not many clinical studies have been conducted for drug research and discovery because of its high potential of interference with the CVS. Therefore, our laboratory uses rat models to study the role of eNOS within specific regions of the brain on cardiovascular responses elicited by pain. We have investigated the effects of eNOS blockade within the dlPAG on cardiovascular responses and neurotransmission during mechanical, heat, and cold nociception. Our results show that eNOS antagonism within the dlPAG augments cardiovascular responses during heat-induced thermal stimulation [64]. In contrast, antagonism of eNOS activity plays no role in modulating cardiovascular responses during mechanical nociception, and surprisingly, cold-induced thermal stimuli evoke depressor and bradycardic responses that were reversed by the blockade of eNOS within the dlPAG [64]. The following figure shows the changes in MAP and HR during all the three types of pain stimuli before and after the administration of an eNOS antagonist (Figure 3, Figure 4 and Figure 5).

Our data provide further insight into the interactions between nociceptive pathways and the CVS involving the eNOS protein within the dlPAG. However, future research using specific NOS isoform agonists or antagonists should continue for the development of potential therapeutic agents for the treatment of neuropathic and other pain with minimal effects on the CVS. To sum it all, there is an old adage that says: “*No pain, no gain*”, which is why we still do not know the exact mechanisms, pain pathways, neurotransmitters, and medications that are relevant to nociception.

This review elaborates on the past and present discoveries regarding the complex relationships of eNOS isozymes, CVS, and nociception. The following are the highlights of our review:

1. The presence of eNOS in dorsal root ganglia and other nervous tissues has been shown to play a role in nociception.
2. The eNOS pathway can be activated by the stimulation of nociceptors.
3. Through its vasodilatory effect, eNOS may affect nociception by regulating the movement of important mediators across the blood vessel walls.
4. Endothelial NOS have pro- or anti-nociceptive effects.
5. Endothelial NOS can also affect nociception through its formation of peroxynitrite and its interaction with TRPV1 as well as with pro- and anti-inflammatory mediators.
6. MAP and HR are both altered during mechanically, heat- and cold-induced nociception. Inhibition of eNOS in PAG affects MAP and HR responses to heat- and cold- but not to mechanical-induced nociception.

## 5. Conclusions and Future Direction

We have discussed the neuroprotective and neurotoxic mechanisms of eNOS in regulating homeostasis during nociception. Notably, mechanically, heat-, and cold-induced nociception produce changes in MAP and HR. While mechanical and heat pain stimuli increase MAP and HR, cold pain stimulus decreases them. Mechanically induced nociception also regulates cardiovascular responses by influencing glutamatergic and GABAergic neurotransmission. Furthermore, NO derived from eNOS within the dlPAG has a potential impact on attenuating the cardiovascular responses during heat-induced nociception, but not mechanically or cold-induced nociception. However, we have not examined the correlations of eNOS activity levels and the aforementioned nociceptive-triggered cardiovascular responses. A closer investigation of these correlations may provide us with a better drug development approach in targeting eNOS to reduce pain, particularly in determining the proper dosage amount to achieve the therapeutic effects and avoid the adverse effects.

The supposedly contradictory effects of nitric oxide in nociceptive pathways, namely the mechanisms that allow for both pro-nociceptive and anti-nociceptive outputs, have been delineated in several scientific studies that aim to explain the disparity [65]. This may be explained by a duality in eNOS function, where it plays a neuroprotective role in earlier stages of ischemia or shear stress versus a neurotrophic role in later stages [3]. In conclusion, this review discusses many roles of eNOS in both physiological and pathological states of the CVS that are related to the eNOS inflammation cascade during experiences of nociception. Moreover, we extensively discuss the interrelated relationship between eNOS, the CVS, and the CNS. We explain the pathogenesis of several cardiovascular diseases, such as hypertension and atherosclerosis, that may happen because of disrupted eNOS activity. We also review the function of eNOS during nociception and how several pharmaceutical products, such as enalapril, atorvastatin, and L-NG-methylarginine hydrochloride (546C88), may modulate the pain pathways.

The management of pain remains a major clinical challenge. A gradual loss in effectiveness after repeated use of opioid drugs, a phenomenon known as opioid tolerance, often develops along with physical dependence. The use of adjuvant non-opioid, particularly ketamine, is known to reduce tolerance and physical dependence. The multimodal approach to pain management, i.e., combining multiple agents from different drug classes, has become increasingly more favorable [66]. Other drugs that can be used in the multimodal approach of pain management include non-steroidal anti-inflammatory drugs (NSAIDs), gabapentinoids, acetaminophen, local anesthetics, and α_2_ agonists. The discoveries of novel non-opioid adjuvants will undoubtedly help mitigate the epidemic of the opioid crisis [67]. Therefore, future research should be geared toward developing novel analgesic agents that target eNOS without significant adverse reactions. These adverse reactions will likely be related to the effects of eNOS on CVS. Our review provides a unique perspective of how eNOS may serve as a potential analgesic target through its complex interaction with nociception and CVS.

## Data Availability

The data presented in this study are available on request from the corresponding author.
